# Describing the genetic architecture of epilepsy through heritability analysis

**DOI:** 10.1093/brain/awu206

**Published:** 2014-07-25

**Authors:** Doug Speed, Terence J. O’Brien, Aarno Palotie, Kirill Shkura, Anthony G. Marson, David J. Balding, Michael R. Johnson

**Affiliations:** 1 UCL Genetics Institute, University College London, London WC1E 6BT, UK; 2 The Departments of Medicine and Neurology, The Royal Melbourne Hospital, The University of Melbourne, Australia; 3 Institute for Molecular Medicine Finland (FIMM), University of Helsinki, Finland; 4 The Broad Institute of MIT and Harvard, Cambridge, USA; 5 Department of Medical Genetics, University of Helsinki, Finland; 6 University Central Hospital, Helsinki, Finland; 7 Division of Brain Sciences, Imperial College London, London W6 8RF, UK; 8 Medical Research Council (MRC) Clinical Sciences Centre, Faculty of Medicine, Imperial College London, UK; 9 Department of Molecular and Clinical Pharmacology, University of Liverpool, UK

**Keywords:** epilepsy, association studies, heritability analysis, complex trait prediction

## Abstract

Epilepsy is a disease with substantial missing heritability; despite its high genetic component, genetic association studies have had limited success detecting common variants which influence susceptibility. In this paper, we reassess the role of common variants on epilepsy using extensions of heritability analysis. Our data set consists of 1258 UK patients with epilepsy, of which 958 have focal epilepsy, and 5129 population control subjects, with genotypes recorded for over 4 million common single nucleotide polymorphisms. Firstly, we show that on the liability scale, common variants collectively explain at least 26% (standard deviation 5%) of phenotypic variation for all epilepsy and 27% (standard deviation 5%) for focal epilepsy. Secondly we provide a new method for estimating the number of causal variants for complex traits; when applied to epilepsy, our most optimistic estimate suggests that at least 400 variants influence disease susceptibility, with potentially many thousands. Thirdly, we use bivariate analysis to assess how similar the genetic architecture of focal epilepsy is to that of non-focal epilepsy; we demonstrate both significant differences (*P = *0.004) and significant similarities (*P = *0.01) between the two subtypes, indicating that although the clinical definition of focal epilepsy does identify a genetically distinct epilepsy subtype, there is also scope to improve the classification of epilepsy by incorporating genotypic information. Lastly, we investigate the potential value in using genetic data to diagnose epilepsy following a single epileptic seizure; we find that a prediction model explaining 10% of phenotypic variation could have clinical utility for deciding which single-seizure individuals are likely to benefit from immediate anti-epileptic drug therapy.

## Abstract (precis)

Epilepsy is highly heritable, but its genetic architecture is poorly understood. Speed *et al.* estimate the number of susceptibility loci, show that common variants account for the majority of heritability, and demonstrate that epilepsy consists of genetically distinct subtypes. They conclude that gene-based prediction models may have clinical utility in first-seizure settings.

## Introduction

Epilepsy is a common, serious neurological disease, defined by an enduring predisposition to epileptic seizures (Fisher *et al.*, 2005), which across North America and Europe affects approximately five people in every 1000 (Banerjee *et al.*, 2009). It is a highly heterogeneous condition that encompasses a spectrum of clinical subtypes, defined by EEG, seizure type and brain imaging criteria. Although clinical classifications are constantly evolving and remain a source of debate (Korff and Scheffer, 2013), patients with epilepsy can be divided into one of two broad categories: focal epilepsy, defined as seizures originating within one cerebral hemisphere, and non-focal epilepsy, of which the majority have generalized epilepsy and a smaller proportion are unclassifiable (Berg *et al.*, 2010). Where care has been taken with seizure classification, ∼60% of people with epilepsy are classified as focal (Banerjee *et al.*, 2009).

Although traditional estimates of heritability for epilepsy vary greatly, depending on the method used, the population sampled and the mixture of clinical subtypes considered, studies have consistently demonstrated that the condition has a substantial genetic component; estimates of heritability from twin studies typically fall in the range 25–70% (Miller *et al.*, 1998; Kjeldsen *et al.*, 2001). By contrast, the molecular genetic factors affecting common forms of epilepsy remain poorly understood. The largest genome-wide association study (GWAS) to date, which considered 3445 Caucasian patients with focal epilepsy and 6935 control subjects, found no single nucleotide polymorphisms (SNPs) significantly associated with risk (Kasperavičirūt ė *et al.*, 2010). In a smaller GWAS of 1087 Chinese focal patients and 3444 controls, genome-wide significance was achieved by a single locus on 1q32.1 (minimum *P = *1 × 10^−8^; Guo *et al.*, 2011), whereas a GWAS of 1527 European generalized patients and 2451 controls reported significant loci at 2p16.1 and 17q21.32 (minimum *P = *2 × 10^−9^ and *P = *9 × 10^−9^, respectively; EPICURE Consortium *et al.*, 2012). None of these three loci have yet been replicated in independent studies. For a minority of epilepsy cases, rare copy number variants have been shown to confer risk for both focal and non-focal forms of epilepsy (Dibbens *et al.*, 2009; de Kovel *et al.*, 2010; Heinzen *et al.*, 2010), whereas for uncommon, monogenic forms of epilepsy, many causal genes have been identified using linkage analysis or exome sequencing of parent-offspring trios (Heinzen *et al.*, 2012; Epi4K Consortium *et al.*, 2013; Hildebrand *et al.*, 2013); however, altogether these findings explain only a small fraction of the overall population susceptibility of epilepsy.

To date, GWAS have predominantly focussed on marginal (single-SNP) analyses, where each SNP is tested individually for association with the phenotype. To allow for the large number of SNPs being tested (from a few hundred thousand to many millions), a SNP is only declared associated if its *P*-value is below a stringent significance threshold (typically *P* < 5 × 10^−8^). So although GWAS have successfully discovered SNPs influential for (or which tag variants which influence) a large variety of phenotypes, with standard sample sizes, they will struggle to detect SNPs of moderate or weak effect. In recent years, methods have been developed for assessing the joint influence of multiple SNPs on phenotypes. A major advantage of these methods is that they can appreciate the contribution of variants with effect sizes too small to be picked up through traditional marginal analysis. In particular, it has been shown that by applying SNP-based heritability analysis to GWAS of nominally unrelated individuals, it is possible to estimate the total variance explained by common variants (Yang *et al.*, 2010, 2011; Speed *et al.*, 2012), whereas by using a bivariate extension of the method it is possible to examine the amount of overlap between the genetic architecture of two traits (Lee *et al.*, 2012).

In this study, we use extensions of SNP-based heritability analysis to reconsider the impact on susceptibility to epilepsy of common variants, defined as those with minor allele frequency >0.01, and to describe the genetic architecture of the disease. Our data set consists of genome-wide SNP data for 1258 epilepsy cases and 5129 population controls. Although marginal analysis finds no individual SNPs significantly associated with susceptibility to epilepsy, we determine that collectively common SNPs explain a sizeable proportion of phenotypic variation: 26% [standard deviation (SD) 5%] when considering all epilepsy and 27% (SD 6%) when considering patients with focal epilepsy. These estimates account for inflation due to population structure and genotyping errors. By considering genome-wide distributions of heritability that are consistent with the results from our association and heritability analyses, we show that epilepsy is a highly polygenic trait with a minimum of 400 susceptibility loci, but potentially many thousands, and that the majority of heritability resides with loci which individually explain only a small fraction (<0.04%) of phenotypic variation. These results indicate that large meta-analyses, involving tens of thousands of individuals, will be required to confidently detect individual SNPs influencing susceptibility to epilepsy.

One measure of the genetic similarity between two traits is ρ, the correlation between SNP effect sizes for each trait: ρ = 1 indicates the two traits have identical genetic aetiologies whereas ρ = 0 indicates no overlap. Using bivariate analysis (Lee *et al.*, 2012), we estimate ρ = 0.45 for focal and non-focal epilepsy. This result shows that there are significant differences between the genetic architectures of the two subtypes (*P = *0.004 when testing the hypothesis ρ = 1), reinforcing the belief that focal and non-focal forms of epilepsy represent distinct disorders. However, it also demonstrates a significant overlap between these two subtypes of epilepsy (*P = *0.01 when testing ρ = 0); this suggests there is scope to improve current clinical classifications, perhaps by incorporating genetic markers, which would facilitate the identification of subtype-specific genetic associations. Our result emphasizes the importance of considering both ‘all epilepsy’ and its individual clinical subgroups in the search for susceptibility loci.

Conceptually, epilepsy is said to exist after at least one unprovoked, non-febrile seizure and when there is a high risk of recurrence (Fisher *et al.*, 2005). As ∼50–60% of individuals who experience a first, unprovoked, non-febrile epileptic seizure will never experience a second (Berg *et al.*, 2010), on a practical level the diagnosis of epilepsy has traditionally relied on the presence of at least two epileptic seizures, as then the chance of recurrence is sufficiently high (60–90%; Hauser *et al.*, 1998). Recently, the International League Against Epilepsy (ILAE) have adopted a new definition of epilepsy, such that an individual experiencing their first seizure should be considered to have epilepsy if their probability of further seizures is similar to the general recurrence risk after two seizures (Fisher *et al.*, 2014).

Given the substantial estimates of variance explained, then with sufficient sample size it should be possible to construct a reasonable prediction model for epilepsy using genome-wide SNP data. Although the low prevalence of epilepsy means that this model would have limited value in terms of predicting which individuals in the general population will develop epilepsy, we examine how well they could assist the diagnosis of epilepsy for patients who have experienced a first epileptic seizure. We find that the ability of such a model to predict which single-seizure individuals will have subsequent seizures depends heavily on the distribution of liability values of individuals for whom the first seizure remains an isolated event. In the best case scenario, we determine that to achieve AUC (area under the receiver operating curve) > 0.75, which has been considered a threshold for clinical use (Janssens *et al.*, 2006), it is necessary to construct a prediction model explaining 10% of phenotypic variation.

## Materials and methods

Epilepsy patients were recruited to our study by epilepsy specialists at UK epilepsy centres (Speed *et al.*, 2014). Inclusion criteria for the study were: (i) epilepsy patients aged 5 years or older; (ii) two or more unprovoked, non-febrile seizures; and (iii) able to give informed consent. Exclusion criteria were: (i) provoked seizures (e.g. alcohol); (ii) acute symptomatic seizures (e.g. acute brain injury); and (iii) progressive neurological disease (e.g. brain tumour). Patients were classified according to ILAE guidelines (Commission on Classification and Terminology of the ILAE, 1989; Berg *et al.*, 2010). Epilepsy subtype was determined by clinicians, and classified as focal, generalized or unclassified (unclassified where there was no evidence for focal onset either clinically or from neuroimaging, but where the EEG did not show a generalized epileptic discharge). A breakdown of epilepsy subtypes is provided in Supplementary Table 1.

After quality control, our data comprise 1258 UK epilepsy patients (958 of subtype focal, 151 generalized and 149 unclassified), combined with 5129 controls (2655 from the 1958 Birth Cohort and 2464 from the National Blood Service; The Wellcome Trust Case Control Consortium, 2007). Before imputation, individuals were recorded for 299 735 autosomal SNPs with minor allele frequency >0.01; after imputation against the 1000 Genome Phase I June 2011 (interim) reference panel using IMPUTE2 (The 1000 Genomes Project Consortium, 2010; Howie *et al.*, 2011), this number increased to 4 238 038. Our quality control steps (detailed in full in the Appendix) sought to exclude suspect samples, poorly genotyped or imputed SNPs, and apparent population outliers. Additionally, we removed close relatedness by filtering samples so that no pair remained with estimated kinship (computed using allelic correlations; Astle and Balding, 2009) >2.6%, a level of relatedness slightly lower than that expected between second cousins (Yang *et al.*, 2010).

Details of our statistical analyses are provided in Appendix 1.

## Results

### Marginal association analysis

**Figure 1:**
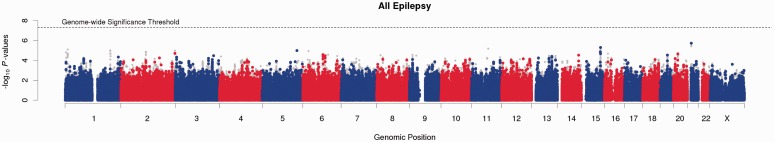
Manhattan plot for single SNP tests of association. Points report -log_10_ *P*-values from single-SNP tests of association for the phenotype all epilepsy (1258 cases, 5129 controls). Red/blue points correspond to genotyped SNPs, grey to imputed. The conventional threshold for genome-wide significance (5 × 10^−8^) is marked by a horizontal dashed line. Manhattan plots for the phenotypes focal, non-focal and generalized epilepsy are provided in Supplementary Fig. 3.

Figure 1 presents *P*-values from marginal tests of association for susceptibility to all epilepsy (1258 cases, 5129 control subjects). We used an additive logistic regression model, including as covariates sex and the five leading principal component axes (the appropriate number of axes to include was determined through heritability analysis, see below and Supplementary Fig. 1). The genomic inflation factor is 1.05. The smallest association *P*-value for any SNP is *P = *1.9 × 10^−6^, considerably above 5 × 10^−8^, the conventional threshold for genome-wide significance. We had 80% power to detect a variant explaining >0.46% of liability variation (see below for an explanation of the liability model) and 50% power to detect a variant explaining >0.35% (Supplementary Fig. 2) We also performed the analysis using only focal epilepsy patients (958 cases), and using only non-focal patients (300 cases consisting of patients with generalized and unclassified epilepsy combined). Again, no SNPs reached genome-wide significance (Supplementary Fig. 3); the smallest *P*-values were *P = *1.9 × 10^−6^ (inflation factor 1.05) and *P = *8.2 × 10^−8^ (inflation factor 1.01), respectively. Our results for focal epilepsy are consistent with those of Kasperavičirūt ė *et al.* (2010), who concluded there was no evidence for common SNPs affecting focal epilepsy susceptibility with odds ratios >1.3 (their study included our control samples). Notably, we found no support for rs2292096 within CAMSAP1 (in our analysis this SNP had *P = *0.26, whereas the minimum *P* across the 178 SNPs within this gene is 0.22). The study identifying this SNP considered focal epilepsy patients of Chinese ancestry (Guo *et al.*, 2011) whereas our patients are of European ancestry. We also consider generalized epilepsy (151 cases), identifying a single locus within SYNRG (top SNP rs116499908, *P = *3.3 × 10^−8^; inflation factor 1.01).

### Variance explained by common single nucleotide polymorphisms

When considering a disease phenotype, it is convenient to suppose an underlying liability model (Supplementary Fig. 4); this assumes that case/control status is determined according to whether or not an individual’s liability, an unobservable, normally distributed random variable, lies above or below a threshold (Falconer and Mackay, 1996). On this scale, estimates of variance explained are invariant to disease prevalence and study ascertainment. To estimate ${\text{h}}_{\text{L}}^{2}$, the proportion of phenotypic liability variation which can be attributed to common SNPs, we first use LDAK (Speed *et al.*, 2012) to calculate a kinship matrix based on allelic correlations across autosomes, using an additive encoding of SNPs, with values centred and scaled to have mean zero and variance one. Then, including as fixed effects sex and the top five principal component axes as used in the association analysis above, we use restricted maximum likelihood (REML) to estimate ${\text{h}}_{{}_{\text{O}}}^{2}$, the fraction of phenotypic variation on the observed scale (cases 1, controls 0) attributable to the kinship matrix. ${\text{h}}_{\text{L}}^{2}$ is then related to ${\text{h}}_{{}_{\text{O}}}^{2}$ via

$${\text{h}}_{\text{L}}^{\text{2}}={\text{ h}}_{\text{o}}^{2}\times {\text{K}}^{\text{2}}{\left(\text{1 }-\text{ K}\right)}^{\text{2}}/\text{ p}\left(\text{1 }-\text{ p}\right){\text{z}}^{\text{2}},$$

where K is the disease prevalence, p is the proportion of cases in the sample, and z is the standard normal density at the liability threshold (Dempster and Lerner, 1950; Yang *et al.*, 2011) (Supplementary Fig. 4). The accuracy of this transformation has been questioned for extreme prevalences (Yang *et al.*, 2011), so to test its appropriateness for our study, we simulate phenotypes with K = 0.005 and *P = *1258/6387 (the values we use when analysing the phenotype all epilepsy). We find that the resulting estimates of ${\text{h}}_{\text{L}}^{2}$ are on average 90% of the true values (Supplementary Fig. 5), indicating that heritability analysis will tend to moderately underestimate the total variance explained on the liability scale for low prevalence diseases.

Table 1 reports our estimates of ${\text{h}}_{{}_{\text{O}}}^{2}$ and ${\text{h}}_{\text{L}}^{2}$ for all epilepsy, and for the subtypes focal and non-focal (generalized and unclassified patients combined). As expected, estimates with imputed SNPs included are larger than those based only on genotyped SNPs, on average by about a quarter. The final column reports ${\text{h}}_{\text{C}}^{2}$, which adjusts the corresponding estimate of ${\text{h}}_{\text{L}}^{2}$ for inflation due to population stratification and genotyping errors (see below). To benchmark estimates of ${\text{h}}_{\text{L}}^{2}$, it is possible to estimate ${\text{h}}_{\text{T}}^{2}$, the total liability heritability for each phenotype from estimates of prevalence and sibling relative risk (Falconer and Mackay, 1996; Wray *et al.*, 2010). Using the values reported by Ottman *et al.* (1998) and Peljto *et al.* (2014), we estimate for all epilepsy ${\text{h}}_{\text{T}}^{2}$ = 32% (95% CI 24–41), for focal epilepsy ${\text{h}}_{\text{T}}^{2}$ = 23% (95% CI 5–43), and for non-focal epilepsy ${\text{h}}_{\text{T}}^{2}$ = 36% (95% CI 15–59). Despite their limited precision, the estimates of ${\text{h}}_{\text{T}}^{2}$ suggest that for each epilepsy phenotype, common SNPs are able to explain the majority of liability heritability, and this conclusion holds for alternative estimates of the population prevalence (Supplementary Table 2).

**Table 1:** Estimates of variance explained by common SNPs

Phenotype	Population	Sibling	Total liability	Genotyped SNPs		Imputed SNPs		
	Prevalence	Relative risk	Heritability, ${\text{h}}_{\text{T}}^{2}$	${\text{h}}_{{}_{\text{O}}}^{2}$	${\text{h}}_{\text{L}}^{2}$	${\text{h}}_{{}_{\text{O}}}^{2}$	${\text{h}}_{\text{L}}^{2}$	${\text{h}}_{\text{C}}^{2}$
All epilepsy (1258 cases)	0.005	3.3 [2.5–4.3]	32 [24–41]	31 (6)	23 (4)	42 (6)	31 (5)	26
Focal (958 cases)	0.003	2.6 [1.2–5.3]	23 [5–43]	33 (6)	27 (5)	41 (7)	33 (5)	27
Non-focal (300 cases)	0.002	4.7 [2.1–10.8]	36 [15–59]	21 (7)	38 (12)	24 (8)	46 (14)	44

For each phenotypes, we report estimates of ${\text{h}}_{{}_{\text{O}}}^{2}$, the percentage of variance explained on the observed scale (cases 1, controls 0), and ${\text{h}}_{\text{L}}^{2}$, the corresponding estimate on the liability scale (standard deviations provided in parentheses). ${\text{h}}_{\text{C}}^{2}$ is obtained from ${\text{h}}_{\text{L}}^{2}$ by subtracting the estimated inflation due to population stratification and genotyping errors (see text). For comparison, ${\text{h}}_{\text{T}}^{2}$, the total liability heritability, is estimated for each phenotype based on the prevalence and reported estimates of sibling relative risk (95% CI shown in square brackets).

The epilepsy cases were genotyped using a lower coverage array than the two wild-type control data sets used for this study (Illumina 660Q as opposed to Illumina 1.2 M), so for the main analysis we imputed case and control cohorts separately. However, we additionally imputed all three cohorts together, starting with the subset of SNPs present on all three arrays. The resulting estimate for all epilepsy was ${\text{h}}_{\text{L}}^{2}$ = 31.7%, almost identical to our previous estimate (${\text{h}}_{\text{L}}^{2}$ = 31.5%), suggesting that with sufficient quality control, separate imputation is not a concern (and generally much faster). When computing kinships, LDAK adjusts for uneven tagging (Speed *et al.*, 2012). For comparison, we also omitted this adjustment, following instead the method of Yang *et al.* (2010). For each phenotype, the resulting estimate of ${\text{h}}_{\text{L}}^{2}$ based on imputed SNPs was lower than that using only genotyped SNPs (Supplementary Table 3), a paradoxical result that demonstrates the importance of adjusting for tagging when performing heritability analysis. An additional benefit of the adjustment is that for the imputed data only ∼7% of the 4.3 million SNPs receive a non-zero weighting, effecting a 14-fold data reduction and speed-up when subsequently estimating variance explained.

### Inflation due to residual familial relatedness, population structure and genotyping errors

The reason that we require individuals to be distantly related is because we want to estimate the variance explained only by causal variation in linkage disequilibrium with common SNPs. By contrast, when closely related pairs of individuals are included, they will tend to share long genomic regions leading to long-range tagging, and also estimates of ${\text{h}}_{\text{L}}^{2}$ will possibly include contributions from shared environmental factors. Additionally, estimates will depend on the degree of relatedness between the sampled individuals, whereas with unrelated individuals estimates should be stable across populations (because linkage disequilibrium tends to be stable). We also wish to avoid population differences between cases and controls; when these are present, then variants that correlate with these differences (for example, SNPs whose allele frequencies vary between populations) will contribute towards estimates of variance explained, whether or not they tag true causal variation. We previously formalized a test to measure inflation of estimates of variance explained due to residual relatedness and population structure (Speed *et al.*, 2012). For the phenotype all epilepsy, we calculate that when including five principal component axes as covariates, ∼9% of our estimate of ${\text{h}}_{\text{L}}^{2}$ corresponds to inflation from these sources (i.e. in absolute terms, our estimate was inflated by ∼3%); the 9% value becomes 8% for focal epilepsy and 1% for non-focal epilepsy (Supplementary Fig. 1). These results indicate that the cases and controls are sufficiently well-matched with respect to population and that estimates of variance explained are not substantially affected by residual relatedness.

Estimates of ${\text{h}}_{\text{L}}^{2}$ can also be inflated by genotyping errors, and strict quality control is required when analysing binary outcomes for which cases and controls have been genotyped separately (Yang *et al.*, 2011; Speed *et al.*, 2012). In Appendix 1, we derive a formula for how the heritability of a binary trait changes according to relabeling or exclusion of samples. When two or more control data sets are present, this formula allows us to estimate inflation due to genotyping errors within control individuals; we provide proof through simulation (Supplementary Fig. 6). For ‘all epilepsy’, we estimate that inflation due to genotyping errors in controls accounts for ∼8% of our estimate for ${\text{h}}_{\text{L}}^{2}$ (in absolute terms 2%); for focal epilepsy this figure is 11%, for non-focal epilepsy 3% (Supplementary Table 4). Although having only one case data set prevents us from measuring inflation due to genotyping errors among case individuals, given that the same quality control steps were used, we expect this to be of similar magnitude. Our final estimates of ${\text{h}}_{\text{L}}^{2}$, with these three sources of inflation discounted, are ${\text{h}}_{\text{C}}^{2}$ = 26% for all epilepsy, ${\text{h}}_{\text{C}}^{2}$ = 27% for focal epilepsy and ${\text{h}}_{\text{C}}^{2}$ = 44% for non-focal epilepsy.

### Variance explained by reported susceptibility loci

The methodology above can also be used to estimate the variance explained by a subset of SNPs, by using only these SNPs when constructing the kinship matrix. Firstly, we consider the 6003 SNPs within 500 kb of rs2292096 (located at 1q32.1), rs13026414 (2p16.1) and rs72823592 (17q21.32), which correspond to the three loci identified through previous GWAS (above). The estimate of ${\text{h}}_{\text{L}}^{2}$ is <0.05%, regardless of which phenotype we consider, and in all cases not significantly different from zero (Supplementary Table 5). Secondly, we identify a list of 85 genes implicated in previous epilepsy studies by searching the UniProtKB database (http://www.uniprot.org) using the keyword ‘epilepsy’. Including the 119 630 SNPs located inside or within 20 kb of the transcription start or end sites of these genes, we estimate ${\text{h}}_{\text{L}}^{2}$ = 3.9% (SD 1.0%) for the phenotype all epilepsy; this estimate of variance explained is both significantly greater than zero (*P* < 10^−4^) and also significantly larger than expected given the number of SNPs involved (permuted *P* < 0.01; Supplementary Table 5), indicating that these genes do harbour susceptibility loci, but that collectively they account for a relatively small fraction of the total heritability of epilepsy.

### Estimating the number of causal variants

We explain how it is possible to gain insights into the number of causal variants underlying a phenotype by considering possible ways the total variance explained by common SNPs is distributed across the genome. By causal variant, we mean any source of genetic variation which affects the phenotype (here, epilepsy susceptibility). As we are considering variation tagged by common SNPs, we expect most of these causal variants to also be common SNPs, but this is not necessarily the case because common SNPs will to some extent tag other sources of variation, such as rare variants and copy number variations (see the ‘Discussion’ section).

**Figure 2:**
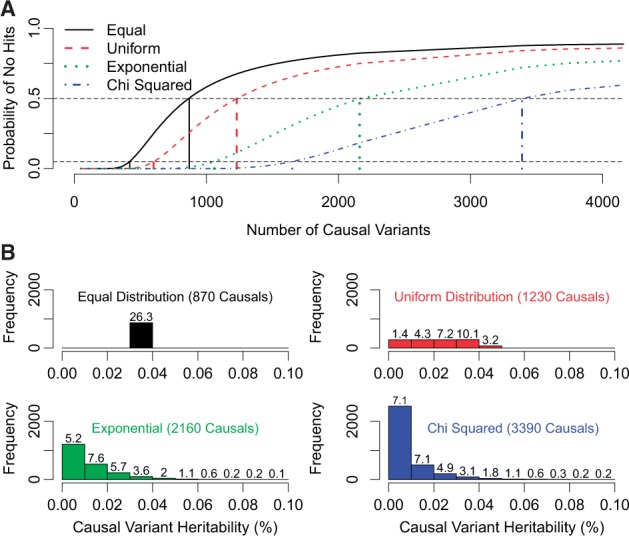
Estimating the number of causal variants. We suppose heritability is distributed over causal variants either equally (black), uniformly (red), exponentially (green) or χ^2^ (blue). (**A**) As the number of causal variants increases (*x*-axis), the average heritability of each variant decreases, and the probability of single-SNP analysis finding no significant associations increases (*y*-axis). For each distribution, our point estimates (lower bounds) for the number of causal variants are the numbers required for this probability to exceed 0.5 (0.05), and are marked by vertical lines. Based on the point estimates, the histograms in **B** show for each distribution how much heritability each causal variant explains. The values above bars report the proportion of variance explained by causal variants within each tranche.

For Fig. 2, we base calculations on the results of our association and heritability analysis for the phenotype all epilepsy; see Supplementary Fig. 7 for focal and non-focal epilepsy. We fix the total variance explained by all causal variants at 26%. For Fig. 2A, we vary the number of causal variants (*x*-axis) and the distribution of heritability across these variants (colour), then calculate the probability that no single variant achieves *P* < 1.9 × 10^−6^ from single-SNP association analysis (Appendix 1). For each distribution, we record how many causal variants are required for this probability to exceed 0.5 (our point estimate) or 0.05 (our estimated lower bound). The most parsimonious scenario is that all causal variants contribute equal heritability (black line), similar to the distribution so far observed for human height and schizophrenia (Kemper *et al.*, 2012), which would suggest 870 causal variants, with a minimum of 420. If we instead suppose the distribution of heritability is uniform (red line), 1230 causal variants are required (minimum 600); if exponential (green line), the distribution considered by Goldstein (2008), 2160 are needed (minimum 1060); if ‘χ^2’^ (a gamma distribution with shape parameter 0.5; blue line), the distribution that applies to heritability contributions if effect sizes are Gaussian (Yang *et al.*, 2010; Speed *et al.*, 2012), the number rises to 3390 (minimum 1650).

Given the point estimates for the number of causal variants for epilepsy, the histograms in Fig. 2B show the spread of heritability for each of the four distributions. Regardless of the distribution considered, the majority of total heritability is accounted for by variants that each explain <0.04% of phenotypic variation. Table 2 shows how the expected success of an epilepsy GWAS depends on its total sample size *n*. For example, if heritability is distributed exponentially with 2160 causal variants, and *n* is increased to 20 000, 50 000 or 100 000 (maintaining a similar case-control ratio) we would expect to detect 6, 103 or 401 of the 2160 causal variants, explaining in total 0.3%, 4.3% or 12.2% of liability variation. The figures are similar regardless of the assumed distribution, and in all cases, >12 500 samples are required before we can expect to find at least one causal variant, although the predicted success can be improved by increasing the ratio of cases to controls (Supplementary Table 6).

**Table 2:** Expected success of single-SNP analyses

**Equal, 870 Causal loci**	*n* = 6387	*n* = 12 500	*n* = 20 000	*n* = 50 000	*n* = 100 000
Expected number of associations	0.1	0.6	3.9	147	710
% of variance explained	0.0	0.0	0.1	4.4	21.4
**Uniform, 1230 Causal loci**	*n* = 6387	*n* = 12 500	*n* = 20 000	*n* = 50 000	*n* = 100 000
Expected number of associations	0.1	0.7	4.6	142	570
% of variance explained	0.0	0.0	0.2	4.9	17.8
**Exponential, 2160 Causal loci**	*n* = 6387	*n* = 12 500	*n* = 20 000	*n* = 50 000	*n* = 100 000
Expected number of associations	0.1	0.9	5.7	103	401
% of variance explained	0.0	0.0	0.3	4.3	12.2
**Chi Squared, 3390 Causal loci**	*n* = 6387	*n* = 12 500	*n* = 20 000	*n* = 50 000	*n* = 100 000
Expected number of associations	0.1	0.9	5.9	96	360
% of variance explained	0.0	0.1	0.3	4.1	11.1

For each assumed distribution of heritability across causal variants, using the corresponding point estimates for the number of causal variants, we estimate the expected number of causal variants detected and the total proportion of liability variation these explain, for different total sample size *n*. We assume the case-control ratio remains fixed at 1258:5129.

### Overlap between subtypes

We can measure the concordance between focal and non-focal epilepsy by ρ, the correlation between the SNP effect sizes for each phenotype; ρ close to 1 indicates that the effect sizes for focal epilepsy are very similar to those for non-focal epilepsy, and would suggest that the clinical divide of patients into focal and non-focal has very little genetic basis. Using a bivariate extension to heritability analysis (Lee *et al.*, 2012), we estimate ρ = 0.45, a value significantly lower than 1 (*P = *0.007 or *P = *0.00002, depending on how the control datasets are matched with focal and non-focal cases; Supplementary Table 7), indicating that patients with focal epilepsy are genetically distinct. However, our estimate of ρ is also significantly greater than 0 (*P = *0.02 or *P = *0.001), indicating that many causal variants are common to both subtypes. These findings suggest that clinical classifications could be improved upon and that at present when conducting an epilepsy GWAS it is advisable to analyse epilepsy subtypes both separately and together.

### Prediction models based on common single nucleotide polymorphisms

We investigated the potential for predicting an individual’s risk of developing epilepsy using a linear prediction model constructed from common SNPs. This model takes the form:

$${\text{G}}_{\text{i}}=\Sigma_{\text{i}}\beta_{\text{j}}{\text{X}}_{\text{ij}}$$

where G_i_ is the predicted risk score for Individual i, X_ij_ is the genotypic value of Individual i for SNP j, and β_j_ is the corresponding SNP effect size. The performance of a prediction model can be measured by ${\text{r}}_{\text{L}}^{\text{2}}$, the squared correlation between each individual's predicted risk G_i_ and their liability value (${\text{r}}_{\text{L}}^{\text{2}}$ can be computed from ${\text{r}}_{{}_{\text{O}}}^{\text{2}}$, the squared correlation between G_i_ and case/control status, using the transformation above). For prediction models constructed from common SNPs, ${\text{h}}_{\text{L}}^{2}$ represents an upper bound on ${\text{r}}_{\text{L}}^{\text{2}}$; how close performance will be to this upper bound in practice will depend on how accurately the effect sizes can be estimated, which in turn depends on the available sample size, the genetic architecture of the trait, and the performance of the estimation method used. Even if we constructed a prediction model with ${\text{r}}_{\text{L}}^{\text{2}}$ close to ${\text{h}}_{\text{L}}^{2}$, due to the low overall prevalence of epilepsy, this model would still have limited use in identifying individuals from the general population likely to develop epilepsy. For example, a prediction model with ${\text{r}}_{\text{L}}^{\text{2}}$ = 0.3 would have AUC 0.89 (Supplementary Fig. 8), but assuming a population prevalence of 0.005, then of the 10% (1%) of individuals with highest predicted risk, only 3.2% (9.6%) would be expected to develop epilepsy.

**Figure 3:**
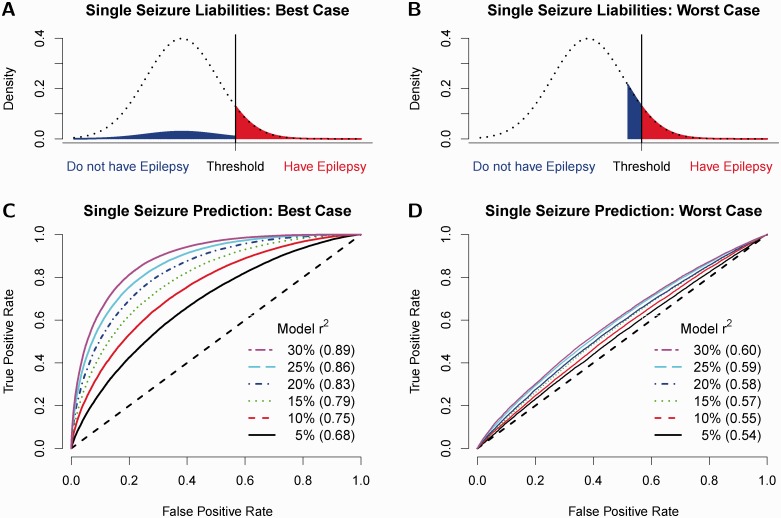
Performance of prediction models for single-seizure patients. **A** and **B** illustrate the two extreme cases for the distribution of liabilities for single-seizure individuals who do not have epilepsy. In the ‘Best Case Scenario,’ their liability distribution matches that of population controls, while in the ‘Worst Case Scenario,’ their liabilities lie just below the case/control threshold. **C** and **D** show, again for the Best and Worst Case Scenarios, how the receiver operating curve depends on the proportion of variance explained by the prediction model (varied between 5% and 30%, indicated by line colour); the AUC (area under receiver operating curve) for each line is provided in parentheses.

By contrast, for individuals who experience a single, unprovoked, non-febrile seizure, the prevalence of epilepsy is much higher (40–50%; Berg *et al.*, 2010). We therefore explore how well a SNP-based prediction model can distinguish single-seizure individuals who have epilepsy (i.e. those destined to have further seizures) from those who do not have epilepsy (i.e. for whom the first seizure remains an isolated event). A major factor influencing the success of our prediction model is the distribution of liabilities of non-recurrent individuals. Figure 3A and B show the ‘Best’ and ‘Worst’ case scenarios. Prediction will be best when the liability distribution for single-seizure individuals without epilepsy matches that of the general population (i.e. that of individuals who never experience any seizures), as then the difference between the average liability values for single-seizure individuals with and without epilepsy will be greatest. Conversely, the scenario where single-seizure individuals without epilepsy have liabilities just below the case/control threshold will prove most challenging.

For the best case scenario, Fig. 3C shows how prediction accuracy depends on ${\text{r}}_{\text{L}}^{\text{2}}$. To achieve AUC 0.75, ${\text{r}}_{\text{L}}^{\text{2}}$ = 0.1 is required (green line). Given such a model, if we were to pick the 10% (20%) of individuals with highest predicted risk, we would expect 80% (75%) of these to experience a second seizure, and we would have identified ∼18% (33%) of the individuals that will subsequently develop epilepsy. However, under the worst case scenario, performance is much poorer, and even a prediction model with ${\text{r}}_{\text{L}}^{\text{2}}$ = 0.3 would only achieve AUC 0.60 (Supplementary Fig. 8). These results demonstrate the need to recruit to studies individuals who experience a single seizure but do not develop epilepsy, as then it will be possible to investigate which liability distribution is most appropriate, and therefore be more precise about the potential success of prediction models in diagnosing epilepsy following a single epileptic seizure.

## Discussion

We have shown how knowledge of ${\text{h}}_{\text{L}}^{2}$, the total proportion of liability variation explained by common SNPs, tells us a large amount about the genetic architecture of the trait under consideration. Although we have focused on epilepsy, the same techniques can be used to improve our understanding of any disease. We have estimated that ${\text{h}}_{\text{L}}^{2}$ is at least 26% for the phenotype all epilepsy and 27% for focal epilepsy, in both cases indicating that common SNPs explain the majority of heritability. By combining results from association and SNP-based heritability analysis, we have provided evidence that epilepsy has many hundreds of susceptibility loci, and that studies comprising tens of thousands of samples and examining the genome in higher resolution (e.g. through full sequencing or via imputation against more comprehensive reference panels) will be required before we can expect to discover a reasonable number of susceptibility loci through single-SNP analyses.

Heritability analysis can also be used to investigate the genetic concordance between subtypes. We have demonstrated that focal and non-focal epilepsy have distinct genetic architectures, reinforcing the view that epilepsy is a genetically heterogeneous trait and that association analyses should take account of clinically defined subtypes; but at the same time, we also found significant overlap between the two subtypes, meaning there is room for subtype definitions to be improved. Ideally, we would have considered subtypes more specific than the two broad categories considered here, but this was not feasible with the sample size available, thus emphasizing the importance of enhanced scientific collaboration to better understand the relationships between the many clinically defined epilepsy subtypes.

${\text{h}}_{\text{L}}^{2}$ provides an upper bound for ${\text{r}}_{\text{L}}^{\text{2}}$, the performance of linear prediction models based on common SNPs. Although for polygenic traits it is unrealistic to expect to achieve ${\text{r}}_{\text{L}}^{\text{2}}$ very close to ${\text{h}}_{\text{L}}^{2}$ (or, equivalently, ${\text{r}}_{{}_{\text{O}}}^{\text{2}}$ close to ${\text{h}}_{{}_{\text{O}}}^{2}$), with large samples sizes, it should be possible to make reasonable progress. For example, for human height, ${\text{h}}_{{}_{\text{O}}}^{2}$ has been estimated to be 0.45 (Yang *et al.*, 2010), and ${\text{r}}_{{}_{\text{O}}}^{\text{2}}$ = 0.36 has been achieved (Makowsky *et al.*, 2011). If, relatively speaking, we are able to do even half as well for epilepsy, the resulting prediction model would explain ∼10% of liability variation and could be used, along with clinical factors, to identify which single-seizure patients are at high risk of experiencing subsequent seizures, therefore satisfying the risk-based definition of epilepsy recently adopted by the ILAE, and are likely to benefit from immediate treatment with anti-epileptic medication.

We have taken care to avoid overestimating ${\text{h}}_{\text{L}}^{2}$. In particular, we recognize that even with stringent quality control, genotyping errors can inflate estimates; for this reason, we have proposed a way to assess and adjust for this effect that we recommend using alongside an existing check for inflation due to population structure and residual relatedness (Speed *et al.*, 2012). There are many factors that might lead to underestimation of variance explained. For example, for prevalences similar to that of epilepsy, we have found that the liability transformation, relied upon for converting values between the observed and liability scale, can result in underestimation of ${\text{h}}_{\text{L}}^{2}$ by about a tenth. A more subtle effect comes from the implicit assumption that the population controls are epilepsy-free. However, even supposing 0.5% of controls (26 individuals) actually have epilepsy, we show that our estimate of ${\text{h}}_{\text{L}}^{2}$ would be only 1 − (1 − 0.005)^2 ^≈ 1% lower than the true value (see Appendix 1 and Supplementary Fig. 9). Imputing genotypes increased our estimate of ${\text{h}}_{\text{L}}^{2}$ by about a quarter. However, despite the high coverage of the 1000 Genome reference panel, it remains that some causal variation will be missed or only partially tagged.

Finally, it should be remembered that while we have focused on common SNPs, these will partially tag rare causal variants, and so our estimate of ${\text{h}}_{\text{L}}^{2}$ will include a contribution from these (Dickson *et al.*, 2010). Similarly, although we assume a linear model, we can still detect additive components of effects that are dominant or epistatic (Zuk *et al.*, 2012). However, for many applications, how much of ${\text{h}}_{\text{L}}^{2}$ is truly attributable to common variation, rather than rare variants and epistasis, is of little importance. For example, our ability to detect a rare causal variant through a GWAS depends not on how much variance the causal variant explains directly, but on the variance explained by the best tagging common SNP, and likewise for loci harbouring interactions. Similarly, the success of a prediction model is how much variation it explains, not the accuracy of the individual effect size estimates.

## Supplementary Material

Supplementary Data

## Abbreviations

GWAS: genome-wide association study
ILAE: International League Against Epilepsy
SNP: single nucleotide polymorphism

## Appendix 1

### Details of methods and quality control steps

#### Estimating the number of causal variants

${\text{h}}_{{}_{\text{O}}}^{2}$ indicates the proportion of phenotypic variance explained (on the observed scale) by common SNPs. For each source of causal variation, let ${\text{h}}_{\text{j}}^{\text{2}}$ denote the variance explained by the SNP best tagging this source of variation. We considered four distributions for ${\text{h}}_{\text{j}}^{\text{2}}$: equal, uniform (with lower bound zero), exponential and ‘χ^2^’ (a gamma distribution with shape parameter 0.5). Each distribution was uniquely determined by the number of causal SNPs: given that there are J causal SNPs, we supposed the jth has ${\text{h}}_{\text{j}}^{\text{2}}$ = F^−1^(j / (1 + J), where F is the cumulative density function of the assumed distribution (however, we checked that results were very similar if instead values of ${\text{h}}_{\text{j}}^{\text{2}}$ were drawn at random from the distribution). Given ${\text{h}}_{\text{j}}^{\text{2}}$, the corresponding test statistic from standard linear regression has a χ^2^ distribution with non-centrality parameter *n*${\text{h}}_{\text{j}}^{\text{2}}$ /(1 − ${\text{h}}_{\text{j}}^{\text{2}}$), where *n* is the sample size. Knowing this, we can calculate *P*_j_, the probability that the SNP surpasses a given significance threshold, and in turn *P** = ∏(1 − *P*_j_), the probability that no SNP achieves significance. Note that we have assumed a model where each causal variant is tagged by only one SNP, when in reality each is likely to be tagged multiple times. Therefore, we are likely to under-estimate *P*_j_ (albeit only slightly, as the set of SNPs tagging each causal variant will be highly correlated), and in turn will tend to under-estimate the number of causal variants required to achieve *P** > 0.5 or *P** > 0.05.

#### Performance of prediction models

We generated prediction models empirically. For example, to construct a prediction model explaining 10% of liability heritability, we first sampled predicted values, G_i_, for 1 000 000 individuals from a Gaussian distribution with mean 0 and variance 0.1, then generated liability values, L_i_, by adding to each G_i_ a Gaussian error term with mean 0 and variance 0.9. Individuals with L_i_ > Ф^−1^(0.995) = 2.58 were cases, the remainder were controls (Ф represents the cumulative density function of the standard normal distribution). To consider population prediction, all individuals were considered. To consider prediction for single-seizure individuals, we focused on a subset containing 45% cases (the average proportion of single-seizure individuals who will experience a second seizure; Berg *et al.*, 2010). For the best case scenario, we picked controls at random; for the worst case scenario, the controls were individuals with Ф^−1^(0.99) < L_i_ < Ф^−1^(0.995), i.e. liability values just below the case/control threshold.

#### Dilution of heritability

Suppose for a trait that the total proportion of phenotypic variance explained by common SNPs is h^2^. We show theoretically in the Supplementary material and through simulation in Supplementary Fig. 9, that if a proportion p of controls have been wrongly labelled as cases, and a proportion p' of cases are in fact controls, then the estimate of variance explained will reduce to (1 − p − p')^2^ h^2^.

#### Inflation due to genotyping errors

Suppose the control samples come from two datasets, of sizes n_1_ and n_2_. In Supplementary Note 2, we show that the estimate of total variance explained can be written as

(1) $$h^{\text{2}}=\frac{n_{A}n_{U}}{n^{\text{2}}}{\left(T+\frac{n_{\text{1}}^{\text{2}}}{n_{U}^{\text{2}}}G_{\text{1}}+\frac{n_{\text{2}}^{\text{2}}}{n_{U}^{\text{2}}}G_{\text{2}}\right)}^{\text{2}}$$

where T denotes the true proportion of phenotypic variance explained, while G_1_ and G_2_ correspond to inflation due to genotyping errors within control data sets 1 and 2. It follows that if we were to perform heritability analysis using only control data set 1 and cases, then using only control data set 2 and cases, the corresponding estimates of variance explained are expected to equal

$$h_{1}^{2}=\frac{n_{A}n_{1}}{{\left(n_{A}+n_{1}\right)}^{2}}\left(T+G_{1}\right)\;\text{and}\;h_{2}^{2}=\frac{n_{A}n_{2}}{{\left(n_{A}+n_{2}\right)}^{2}}\left(T+G_{2}\right)\text{.}$$

Therefore, based on estimates of h^2^, ${\text{h}}_{\text{1}}^{\text{2}}$ and ${\text{h}}_{2}^{\text{2}}$, we are able to estimate T, G_1_ and G_2_, from which our heritability estimate adjusted for genotyping errors within controls is n_A_n_U_T/n^2^. We demonstrate the effectiveness of this approach through simulation in Supplementary Fig. 6. The method can readily be extended to accommodate additional control and case datasets.

### Data quality control

Our raw data set comprised 5667 controls used by The Wellcome Trust Case Control Consortium (2930 from the 1958 Birth Cohort and 2737 from the National Blood Service) and 1485 cases. For cases (genotyped on Illumina 660Q), we excluded samples with average heterozygosity outside [0.281, 0.299] (44 samples removed) or missing values for >2% of genotypes (11 additional samples removed). To check the quality of the Illumina sequencing, we re-genotyped 30 SNPs using a Sequenom array, and excluded samples with three or more mismatches (five additional samples removed). Finally, we excluded 64 further samples that seemed to be duplicates (estimated kinship >0.9 with another sample). For the controls (genotyped on Illumina 1.2 M), we followed the recommendations of the Wellcome Trust, excluding 231 samples from the 1958 Birth Cohort and 236 from the National Blood Service, then checked that no individuals remained with extreme heterozygosity, missingness or evidence for duplication. We next ensured there were no pairs of individuals with estimated kinship >0.1875 (15 samples lost), then removed 36 potential outliers identified through principal component analysis. Finally, to reduce the levels of relatedness to those expected by chance, we filtered 83 samples so that no pair remained with estimated kinship >0.026, the absolute value of the minimum observed (Yang *et al.*, 2010). At this point, there remained 5129 controls and 1298 cases (of which 958 were focal, 151 were generalized and 149 were unclassified, whereas 40 had status unknown and were excluded from subsequent analyses). Further phenotypic details are provided in Supplementary Table 1, and a principal component plot is provided in Supplementary Fig. 10.

Before imputation, we excluded SNPs with minor allele frequency <0.01, call rate <0.95, or *P* > 10^−6^ from a test for Hardy-Weinberg Equilibrium. We imputed against the 1000 Genome June 2011 (interim) reference panel using IMPUTE2 with default parameter values (The 1000 Genomes Project Consortium, 2010; Howie *et al.*, 2011). Before association and heritability analysis, we performed SNP quality control a second time, removing those with (expected) minor allele frequency <0.01, (expected) call rate <0.995, INFO <0.98 or, if a genotyped SNP, r2 <0.95 (the latter two metrics are scores computed by IMPUTE2). These thresholds are much stricter than those typically used for marginal association analysis. However, with heritability analysis, it is necessary to be far more cautious, especially when cases and controls have been genotyped separately, as even slight errors can accumulate over SNPs to produce greatly inflated estimates of variance explained (Yang *et al.*, 2011; Speed *et al.*, 2012). For heritability analysis, we used the remaining 4 238 038 autosomal SNPs, of which 299 735 were directly genotyped. For the association analysis, we additionally considered the 89 281 SNPs passing quality control on chromosome X.
