# Supplementary material for: Describing the genetic architecture of epilepsy through heritability analysis
Source: Brain. 2014 Jul 25;137(10):2680–9. doi: 10.1093/brain/awu206 (PMC4163034; doi:10.1093/brain/awu206)
Supplement: Supplementary Data [file supp_137_10_2680__index.html]

Supplementary Data 

# Describing the genetic architecture of epilepsy through heritability analysis

## Supplementary Data

files

**Files in this Data Supplement:**

- Supplementary Data - pdf file
