# Supplementary material for: Describing the genetic architecture of epilepsy through heritability analysis
Source: Brain. 2014 Jul 25;137(10):2680–9. doi: 10.1093/brain/awu206 (PMC4163034; doi:10.1093/brain/awu206)
Supplement: Supplementary Data [file supp_awu206_brain-2014-00220-File005.pdf]

Describing the genetic architecture of epilepsy through heritability analysis: Supplementary Material

Doug Speed,[1*] Terence J. O'Brien,[2] Aarno Palotie,[3,4,5,6] Kirill Shkura,[7,8] Anthony Marson,[9] David J. Balding[1] and Michael R. Johnson[7*]

[1] UCL Genetics Institute, University College London, United Kingdom
[2] The Departments of Medicine and Neurology, The Royal Melbourne Hospital, The University of Melbourne, Australia
[3] Institute for Molecular Medicine Finland (FIMM), University of Helsinki, Finland
[4] The Broad Institute of MIT and Harvard, Cambridge, USA
[5] Department of Medical Genetics, University of Helsinki, Finland
[6] University Central Hospital, Helsinki, Finland
[7] Division of Brain Science, Imperial College London, United Kingdom
[8] Medical Research Council (MRC) Clinical Sciences Centre, Faculty of Medicine, Imperial College London, United Kingdom
[9] Department of Molecular and Clinical Pharmacology, University of Liverpool, United Kingdom

∗ Corresponding authors doug.speed@ucl.ac.uk and m.johnson@imperial.ac.uk

Contents

Note 1: Dilution of heritability.
Note 2: Inflation due to genotyping errors.

Figure 1: Estimating inflation due to cryptic relatedness.
Figure 2: Power for marginal (single-SNP) analyses.
Figure 3: Marginal (single-SNP) association analyses.
Figure 4: The liability model for diseases.
Figure 5: Transforming heritability between liability and observed scales; proof of principle.
Figure 6: Estimating inflation due to genotyping errors; proof of principle.
Figure 7: Estimating the number of causal variants.
Figure 8: Classification performance of prediction models.
Figure 9: The effect of mislabelling samples; proof of principle.
Figure 10: Principal component plot of samples.

Table 1: Phenotypic breakdown of epilepsy cases.
Table 2: Estimates of prevalence affect estimates of h^2_T and h^2_L.
Table 3: Estimates of variance explained using GCTA.
Table 4: Estimating inflation due to genotyping errors.
Table 5: Variance explained by previously reported susceptibility loci.
Table 6: Expected success of marginal (single-SNP) analyses.
Table 7: Bivariate analysis for focal and non-focal epilepsy.

References

1. Speed, D. *et al.* A genome-wide association study and biological pathway analysis of epilepsy prognosis in a prospective cohort of newly treated epilepsy. *Hum. Mol. Genet.* **1**, 247–258 (2014).

2. Speed, D., Hemani, G., Johnson, M. & Balding, D. Improved heritability estimation from genome-wide SNP data. *Am. J. Hum. Genet.* **91**, 1011–1021 (2012).

3. Lee, J. Y. S., Goddard, M. & Visscher, P. GCTA: a tool for genome-wide complex trait analysis. *Am. J. Hum. Genet.* **88**, 76–82 (2011).

Note 1: Dilution of Heritability.

In linear regression, the estimated regression coefficient is $\beta = \mathrm{Cov}(X,Y)/\mathrm{Var}(X)$, where X denotes the genotypic values of the SNP being tested and Y the phenotypic values. For simplicity, suppose both X and Y have been centred and scaled to have mean zero and variance one. For a binary phenotype comprising n_A cases and n_U controls, Y will take value $Y_A = \sqrt{n_U/n_A}$ if a case, and $Y_U = -\sqrt{n_A/n_U}$ if a control. Therefore,

$$\beta = \frac{\sum X_i Y_i}{n} = \frac{n_A Y_A M_A + n_U Y_U M_U}{n} = \frac{\sqrt{n_A n_U}(M_A - M_U)}{n}$$

where $n = n_A + n_U$, and M_A and M_U are the mean values of X across cases and controls, respectively. The proportion of phenotypic variation explained by this SNP will be $\beta^2 = n_A n_A (M_A - M_U)^2/n^2$, and so the important value is $S = M_A - M_U$, the mean difference in genotypic values between cases and controls. Although, strictly speaking, logistic regression would be more appropriate, for small effect sizes, this linear approximation will suffice.

Now suppose that out of the n_U controls, a proportion p are actually cases, and consider the effect on S. The mean genotypic value for cases will continue to be M_A, but the controls will now have mean $(1-p)M_U + pM_A$, and the observed difference will be $S' = (1-p)(M_A - M_U)$. Therefore, each SNP will (on average) contribute only $(1-p)^2$ as much heritability as it would if cases and controls were correctly labelled, so summed over all SNPs, the estimate of total SNP heritability will also be lower by this fraction. Supplementary Fig. 8 demonstrates this relationship holds for values of p up to 0.25. If, additionally, p' cases are wrongly labelled as controls, then similar calculations demonstrate the heritability will be lower by $(1-p-p')^2$.

Note 2: Inflation due to genotyping errors.

When the n_U control samples come from two datasets, of sizes n_1 and n_2, we show that the estimate of total variance explained can be written as

$$h^2 = \frac{n_A n_U}{n^2}\left(T + \frac{n_1^2}{n_U^2}G_1 + \frac{n_2^2}{n_U^2}G_2\right)^2, \tag{1}$$

where T denotes the true variance explained, while G1 and G2 correspond to inflation due to genotyping errors within Control Datasets 1 and 2, respectively.

Suppose that for SNP X, the true difference in mean genotypic values between cases and controls is $S = M_A - M_U$, and so as shown in Supplementary Note 1, the proportion of phenotypic variance the SNP explains is $n_A n_A S^2/n^2$. If A_1 and A_2 denote the amounts by which genotyping errors within each control dataset increase the mean value of X for each dataset, then M_U will be increased by

$$\frac{n_1}{n_U}A_1 + \frac{n_2}{n_U}A_2,$$

and as a result the variance explained by the SNP now equals

$$\frac{n_A n_U}{n^2}\left(S - \frac{n_1}{n_U}A_1 - \frac{n_2}{n_U}A_2\right)^2.$$

If two centred random variables, U and V, are independent, then $\mathbb{E}((U-V)^2) = E(U^2) + E(V^2)$. Therefore, if we suppose that, for any given SNP, the values of S, A_1 and A_2 are independent and have mean zero (which seems reasonable, especially considering that with relatively few SNPs causal and accurate genotyping, we would expect most values to be zero), then we can write the total heritability in the form given by (1) where T, G_1 and G_2 are the sums across all SNPs of S^2, A_1^2 and A_2^2, respectively.

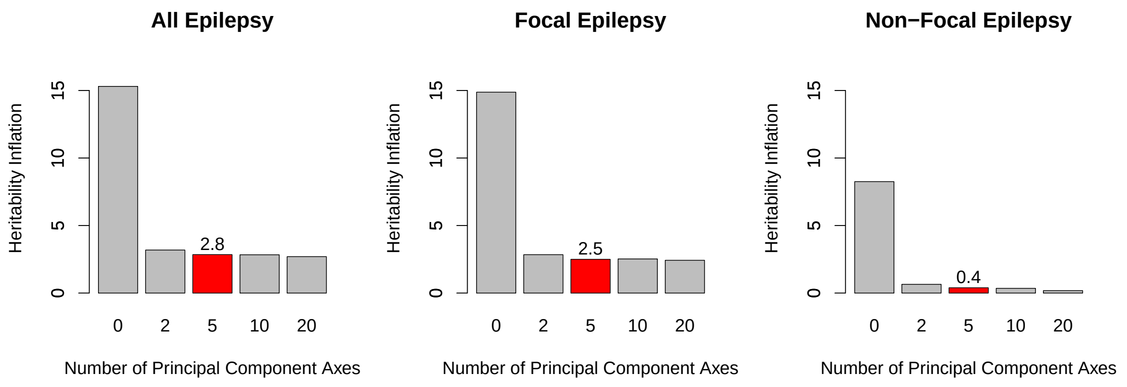

Figure S1. Estimating inflation due to cryptic relatedness. To estimate the extent by which residual familial relatedness and population structure inflates estimates of heritability, we calculate heritability from Chromosomes 1-8, then from Chromosomes 9-22, and from the sum of these two estimates subtract the estimate of heritability from all autosomes. The x-axis indicates the number of principal component axes included in the regression model, the y-axes reports the estimate of inflation (values correspond to the liability scale and are in absolute terms). These tests indicate there is little benefit including more than five principal component axes as fixed effect covariates (red boxes), at which point the estimated inflation is 2.8% for all epilepsy, 2.5% for focal epilepsy and 0.4% for non-focal epilepsy; in relative terms, these values represent 9%, 8% and 1% of the estimate of h^2_L.

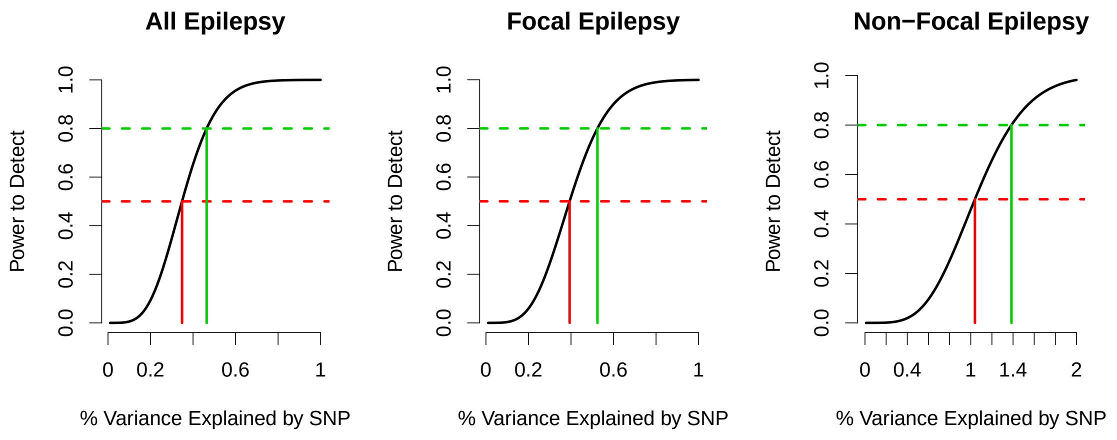

Figure S2. Power for marginal (single-SNP) analyses. The test statistic from standard linear regression has a χ^2 distribution with non-centrality parameter $nh^2/(1-h^2)$, where n is the sample size and h^2 is the proportion of phenotypic variance explained by the SNP. For a binary trait, h^2 corresponds to the observed scale (cases 1, controls 0), but knowing the disease prevalence and study ascertainment, it can be converted to the liability scale using the transformation detailed in Figure S5. The three plots correspond to the phenotypes all epilepsy (1258 cases, 5129 controls), focal epilepsy (958 cases) and non-focal epilepsy (300 cases). In each plot, the y-axis shows how the probability that a SNP achieves genome-wide significance (5×10^{-8}) depends on the proportion of liability variance it explains (x-axis). The red and green lines mark the thresholds for 50% and 80% detection power.

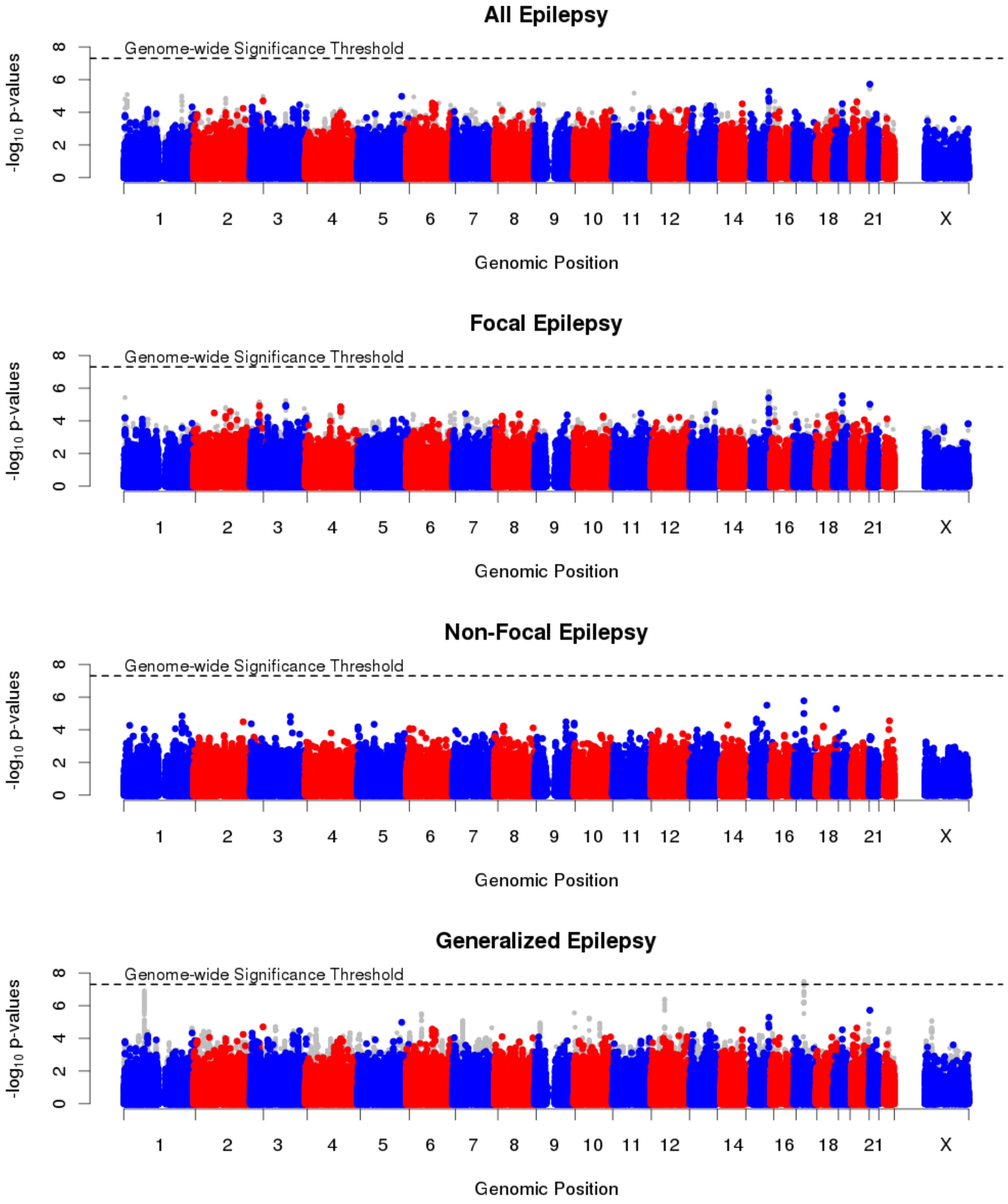

Figure S3. Marginal (single-SNP) association analyses. Each plot reports $-log_{10}$ p-values from marginal tests of association, where red/green points correspond to genotyped SNPs and grey to imputed. From top to bottom, the plots refer to all epilepsy (1258 cases, 5129 controls), focal epilepsy (958 cases), non-focal epilepsy (300 cases) and generalized epilepsy (151 cases). The conventional threshold for genome-wide significance ($5{\times}10^{-8}$) is marked in each plot by a horizontal dashed line.

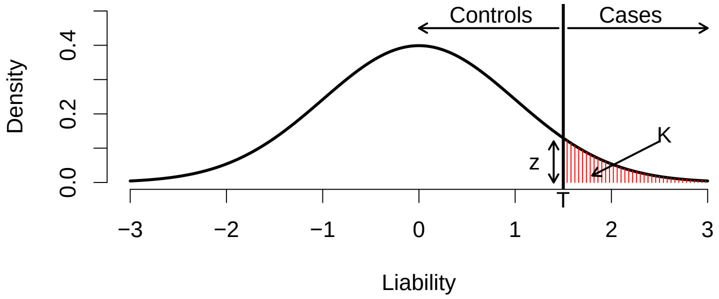

Figure S4. The liability model for diseases. When considering binary outcomes, it is convenient to assume an underlying liability model, whereby case/control status is determined by whether an unobserved random variable (the "liability") is above or below a threshold value, T. The liability is assumed to have a standard normal distribution, so T is determined by the prevalence of the disease K: the area to the right of T must equal K (e.g., for all epilepsy, $K = 0.005$ so $T = 2.58$). z is the height of the standard normal curve at the threshold.

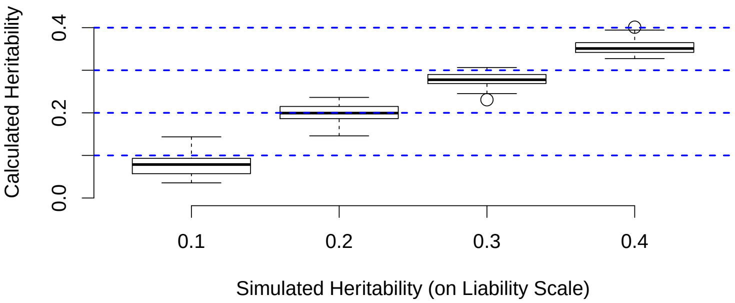

Figure S5. Transforming heritability between liability and observed scales; proof of principle. To convert estimates of heritability between the observed (h^2_O) and liability (h^2_L) scales, we use the formula $h^2_L = h^2_O \times K^2(1-K)^2/p(1-p)z^2$, where K is the prevalence, p the proportion of cases in the sample and z is the standard normal density at the liability threshold. To test the reliability of this transformation for our analysis, we constructed a population dataset consisting of 280 000 individuals genotyped for 20 000 independent SNPs. We generated liability values with heritability 0.1, 0.2, 0.3 or 0.4, supposing 2000 SNPs were causal; individuals with liability $> \Phi^{-1}(0.995)$, were deemed cases, the remainder controls (Φ is the standard normal cumulative density function). From this population dataset, we selected at random 1258 cases and 5129 controls, using which we estimated heritability. On average, estimate of h^2_L based on this subset of samples (each box corresponds to 50 repetitions) are approximately a tenth lower than the true values (horizontal lines).

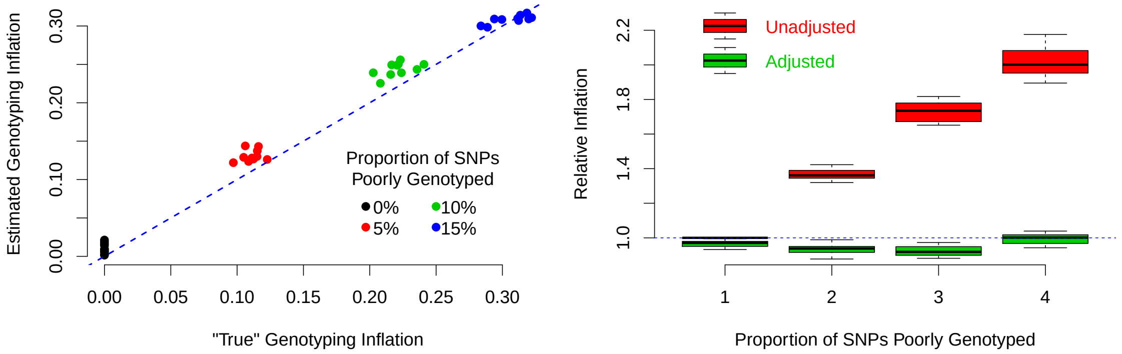

Figure S6. Estimating inflation due to genotyping errors; proof of principle. For this simulation, we used (pre-imputation) genotypes for Chromosomes 1 and 2 (40 032 SNPs) and all 6387 individuals. We generated liability values with 50% heritability supposing 2000 SNPs were causal; individuals with liability $> \Phi^{-1}(0.6)$, became cases, the remainder controls. We first computed a kinship matrix using the observed genotypes, from which we obtained a "correct" estimate of h^2_O. In order to simulate genotyping errors, we randomly divided the control individuals into two sets. For 5% of SNPs, we picked a random error term (drawn from a uniform distribution on [-0.05,0.05]), and added this value to genotypes for individuals within the first control dataset. We did the same for individuals within the second control dataset (again affecting 5% of SNPs). We then recomputed kinships and obtained a new estimate of h^2_O; the difference between this and the correct estimate represents the "true" inflation due to genotyping errors. We also considered increasing the proportion of affected SNPs to 10% and 15%, and repeated the process for a total of 10 phenotypes. For each phenotype, the left plot shows how well we can estimate the inflation due to genotyping errors using the method outlined in the main text. The right plot presents unadjusted (red) and adjusted (green) estimates of h^2_O (all values are relative to the correct estimates). We see that even when genotyping errors more than double the estimate of h^2_O, we can still expect to recover the correct estimate with reasonable accuracy.

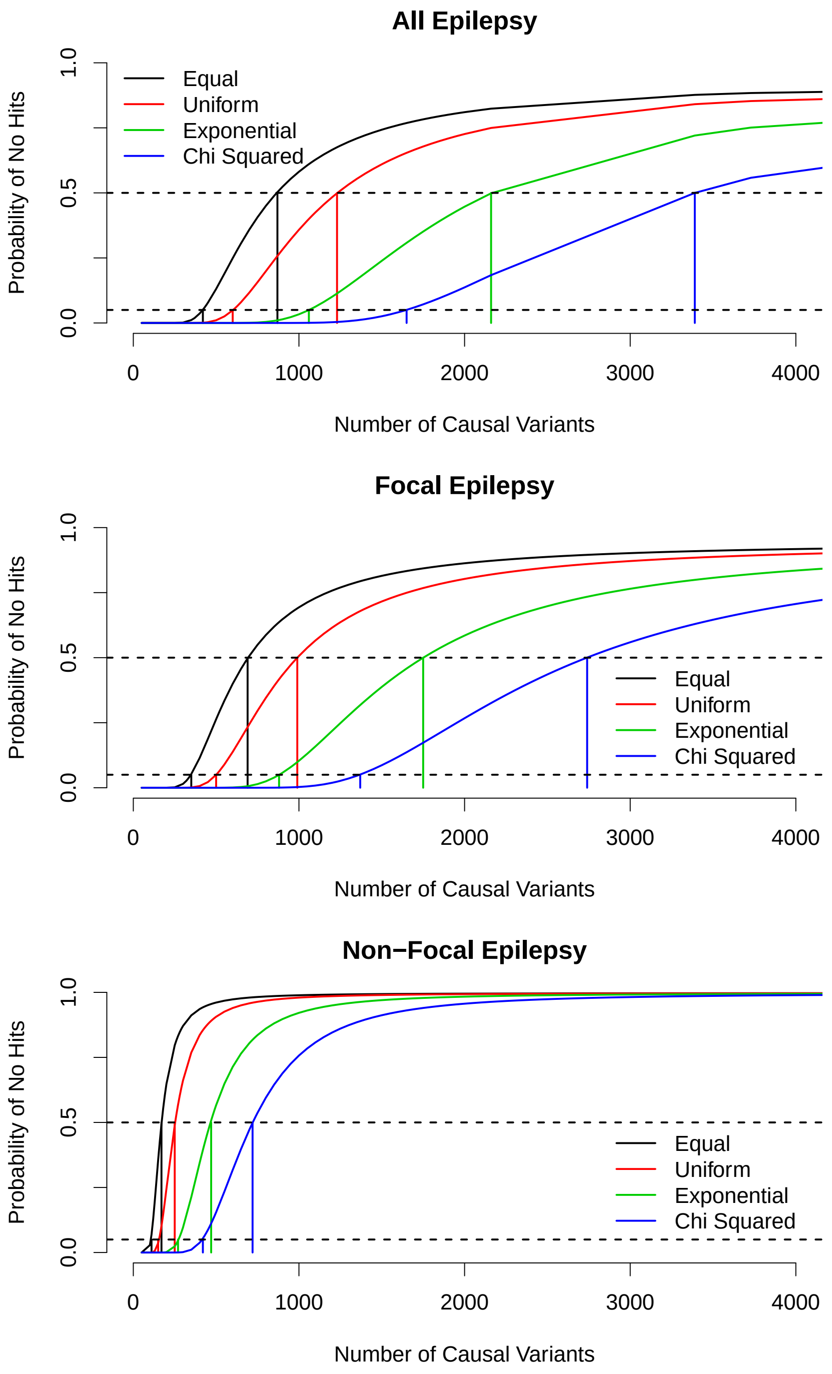

Figure S7. Estimating the number of causal variants. In the main text, we focus on estimating the number of causal variants for the phenotype all epilepsy. Here, we also consider the subtypes focal and non-focal epilepsy. We vary the number of causal variants (x-axis) and the assumed distribution of heritability across these variants (black, equal; red, uniform; green, exponential; blue, chi squared), with parameters chosen to ensure the total variance explained by causal variants matches our estimates based on the real data; for these simulations, the y-axis reports the probability that in marginal tests of association, no SNP exceeds the lowest p-value observed for the real data. For each distribution, our point estimate (lower bound) for the number of causal variants is the number required for this probability to exceed 0.5 (0.05). These estimates are marked by vertical lines.

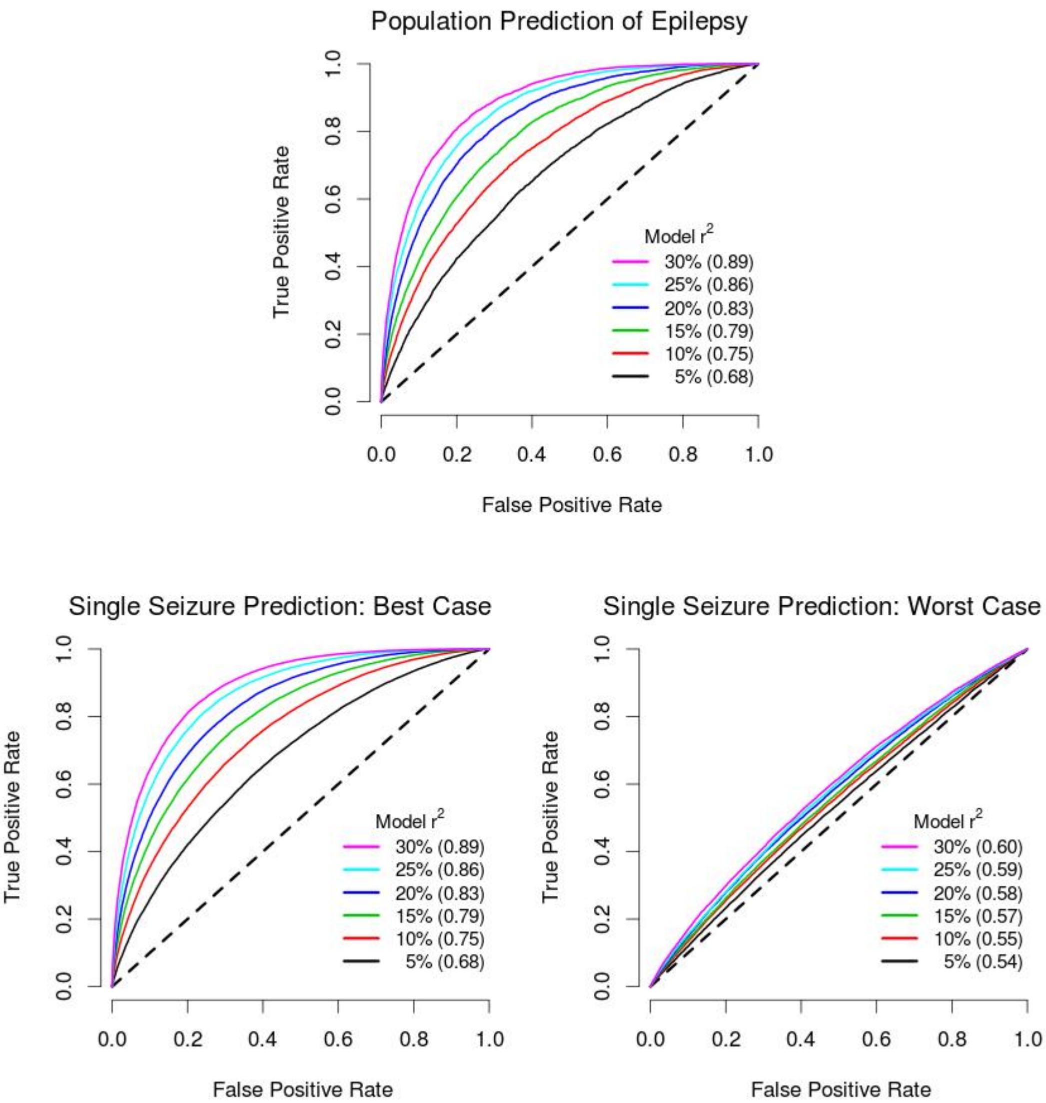

Figure S8. Classification performance of prediction models. Each ROC curve plots the true positive rate (the proportion of individuals who develop epilepsy that are correctly classified) against the false positive rate (the proportion of individuals who do not develop epilepsy but are wrongly classified). We consider different prediction models, defined by the proportion of liability variation each explains (from 5 to 30%, indicated by line colour). The left curve reports the performance of the model applied to the general population; the middle and right curves correspond to single-seizure individuals, assuming either the Best or Worst Case Scenarios for the liability distribution of single-seizure individuals who do not develop epilepsy (see main text). The figures in parentheses report AUC (area under curve) for each model. AUC values are invariant to ascertainment, which is the reason why values match for the first two curves.

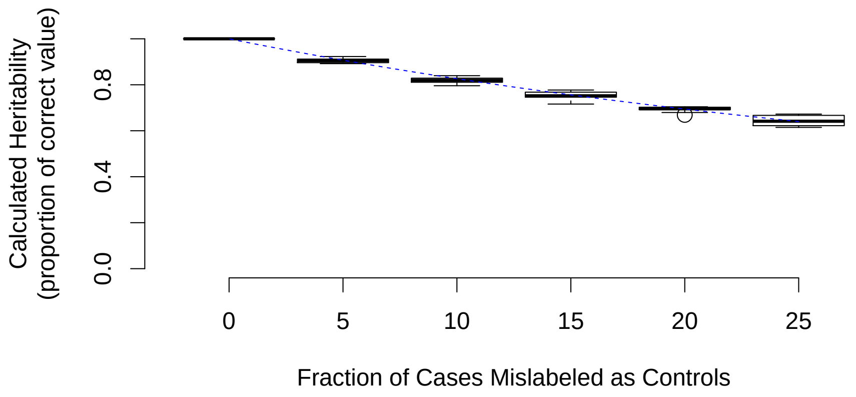

Figure S9. The effect of mislabelling samples; proof of principle. Given that a fraction p of controls are in fact cases, we calculate that the estimate of h_L^2 (or equivalently, that of h_O^2) should be $(1-p)^2$ times the true value. We test this theory through simulation, using (pre-imputation) genotypes for Chromosomes 1 and 2 (40 032 SNPs) and all 6387 individuals. We first generated liability values with 50% heritability supposing 2000 SNPs were causal, according to which we dichotomised individuals into cases and controls based on a threshold of 0. The "correct" heritability estimate is that obtained using the correct labellings. Then we randomly sample 5% of cases, relabel these as controls and re-estimate heritability, recording the average heritability over ten samplings (measured as a proportion of the correct estimate). We also consider 10%, 15%, 20% and 25% of cases wrongly labelled, and repeat for a total of 50 phenotypes. We find that the observed proportions closely concentrate around those expected (dashed line).

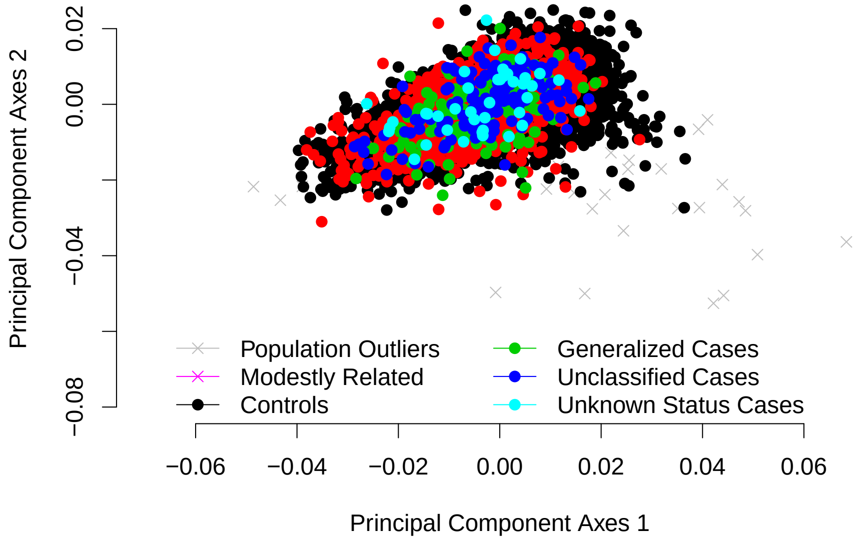

Figure S10. Principal component plot of samples. The x and y-axes correspond to the first two axes from principal component analysis. After checks of genotyping accuracy, and removal of individuals so that no pair remained with estimated kinship > 0.1875, (a level of relatedness halfway between those expected for full-sibs and cousins), 6561 samples remained. Then 36 were excluded as suspected population outliers (grey) and 83 to remove pairwise relatedness higher than that expected by chance (purple). Of the remaining samples, 5129 were controls (black) and 1295 cases, of which 958 had type focal (red), 151 generalized (green), 149 unclassified (dark blue) and 40 had status unknown (light blue).

Epilepsy Subtype	Number of Individuals
Focal: Hippocampal sclerosis lesion	61
Focal: Lesion other than hippocampal sclerosis	148
Focal: Lesion negative	379
Focal: Lesion status not documented	370
Generalized: Childhood absence epilepsy	15
Generalized: Juvenile absence epilepsy	9
Generalized: Juvenile myoclonic epilepsy	48
Generalized: Generalized tonic-clonic seizures	3
Generalized: Other type or type not documented	76
Unclassified	149

Table S1. Phenotypic breakdown of epilepsy cases. All epilepsy cases were carefully phenotyped by epilepsy specialists (see [1] for details). Clinical data included: numbers and types of epilepsy seizures pre-treatment, results of MRI and EEG, information on drug treatment and outcome. Most patients were reviewed on at least ten occasions post starting treatment, and all patients were re-evaluated at data entry to confirm accuracy of seizure and epilepsy classification. From this information it was possible to identify subsets of individuals exhibiting specific epilepsy subtypes, some of which are listed above. However, to ensure reasonable sample size we focused analysis on three broad phenotypes: all epilepsy, focal epilepsy and non-focal epilepsy.

Phenotype	h^2_O	K	h^2_T	h^2_L	K	h^2_T	h^2_L	K	h^2_T	h^2_L
**All Epilepsy**	42 (6)	0.0020	27 [19-33]	26 (4)	0.005	32 [24-41]	31 (5)	0.010	39 [28-50]	37 (6)
**Focal**	41 (7)	0.0012	19 [4-35]	28 (5)	0.003	23 [5-43]	33 (5)	0.006	26 [5-50]	38 (6)
**Non-Focal**	24 (8)	0.0008	30 [13-49]	39 (12)	0.002	36 [15-59]	46 (14)	0.004	41 [18-70]	52 (16)

Table S2. Estimates of prevalence affect estimates of h^2_T and h^2_L. The estimate of total liability heritability, h^2_T, depends the population prevalence, K (as well as on sibling relative risk); similarly, the estimate of variance explained on the underlying liability scale, h^2_L, which is obtained by transforming the corresponding estimate on the observed scale, h^2_O, also depends on K. In the main text, we assumed $K = 0.005$ for the phenotype all epilepsy; here, we consider three different values (0.002, 0.005 and 0.01), continuing to suppose that 60% of epilepsy patients are classified focal, and report for each the resulting estimate of h^2_T (95% confidence interval in parentheses) and of h^2_L (standard deviation in parentheses). Although varying K affects the estimates of h^2_T and h^2_L considerably, it has only a modest effect on h^2_L/h^2_T, the proportion of total liability heritability explained by common SNPs.

				Genotyped SNPs		Imputed SNPs	
**Phenotype**	K	Sibling Relative Risk	h^2_T	h^2_O	h^2_L	h^2_O	h^2_L
**All Epilepsy**	0.005	3.3 [2.5-4.3]	32 [24-41]	21 (5)	16 (3)	18 (4)	14 (3)
**Focal**	0.003	2.6 [1.2-5.3]	23 [5-43]	23 (5)	19 (4)	19 (4)	15 (4)
**Non-Focal**	0.002	4.7 [2.1-10.8]	36 [15-59]	12 (5)	24 (10)	3(5)	6 (9)

Table S3. Estimates of variance explained using GCTA. For the estimates of h^2_L in the main text, we computed a weighted kinship matrix using LDAK [2] which allows for the uneven nature of linkage disequilibrium. Here we report estimates when we omit this adjustment and instead follow the procedure of GCTA, [3] which weights each SNP equally when computing the kinship matrix. We would expect an estimate based on imputed SNPs to be no smaller than that based on genotyped SNPs, but we see that without adjusting for uneven tagging, this proves not to be the case. As before, h^2_T represents the total liability heritability, estimated based on estimates of prevalence and sibling relative risk.

**Phenotype**	Total h^2_L	Common h^2_L	Controls 1 h^2_L	Controls 2 h^2_L	Genotyping Error
All Epilepsy	31	28	0	2	2
All Epilepsy	33	28	3	0	3
All Epilepsy	46	44	0	1	2

Table S4. Estimating inflation due to genotyping errors. For each phenotype, we estimate how much of h^2_L is specific to each control dataset, the sum of which provides an estimate of how much genotyping errors within controls inflate our estimate of h^2_L. The remaining portion of h^2_L is common to all datasets and represents true signal plus inflation due to genotyping errors in cases (with only one case dataset available, it is not possible to separate out the contribution of genotyping errors within cases). Because we restrict that component estimates are non-negative, they do not necessary sum to h^2_L.

**Subset of SNPs**	Phenotype	h^2_L (SD)	$\mathbb{P}_{h^2_L>0}$	Permuted P_1	Permuted P_2
Three susceptibility loci	All epilepsy	0.00 (0.18)	0.50	—	—
	Focal epilepsy	0.04 (0.20)	0.42	—	—
	Non-focal epilepsy	0.00 (0.53)	0.50	—	—
85 susceptibility genes	All epilepsy	3.9 (1.0)	0.00002	0.004	0.002
	Focal epilepsy	3.3 (1.1)	0.001	0.038	0.042
	Non-focal epilepsy	3.5 (2.8)	0.1	0.178	0.182

Table S5. Variance explained by previously reported susceptibility loci. We first estimate the variance explained on the liability scale h^2_L (standard deviation in parenthesis) by the 6003 SNPs within 500 kb of the three loci previously identified through genome-wide association studies: rs2292096 (located at 1q32.1), rs13026414 (2p16.1) and rs72823592 (17q21.32). Next we consider the 119 630 SNPs located inside or within 20 kb of 85 genes obtained by searching the UniProtKB database (http://www.uniprot.org) using the keyword "epilepsy". The first p-value corresponds to a "self-contained" test that the estimate of variance explained is significantly greater than zero. For the susceptibility genes, we additionally provide a p-value for a "competitive test" which assesses whether the 119 630 SNPs explain more variance than expected given the total variance explained by all SNPs. For this we use cyclic permutations to obtain 500 new lists of 119 630 SNPs displaying similar structure to the original list; for each permutation, we increase the SNP index by 2500 (so if the original list contains SNPs 1, 2, 3, ..., the first permutation will consider SNPs 2501, 2502, 2503, ...). Permuted P_1 indicates how often a permutation resulted in a larger estimate of h^2_L; Permuted P_2 indicates how often a permutation resulted in a smaller $\mathbb{P}_{h^2_L>0}$.

Case-Control Ratio 1258:5129

**Equal, 870 Causal Loci**	$n = 6387$	$n = 12500$	$n = 20000$	$n = 50000$	$n = 100000$
Expected Number of Associations	0.1	0.6	3.9	147	710
% of Variance Explained	0.0	0.0	0.1	4.4	21.4
**Uniform, 1230 Causal Loci**	$n = 6387$	$n = 12500$	$n = 20000$	$n = 50000$	$n = 100000$
Expected Number of Associations	0.1	0.7	4.6	142	570
% of Variance Explained	0.0	0.0	0.2	4.9	17.8
**Exponential, 2160 Causal Loci**	$n = 6387$	$n = 12500$	$n = 20000$	$n = 50000$	$n = 100000$
Expected Number of Associations	0.1	0.9	5.7	103	401
% of Variance Explained	0.0	0.0	0.3	4.3	12.2
**Chi Squared, 3390 Causal Loci**	$n = 6387$	$n = 12500$	$n = 20000$	$n = 50000$	$n = 100000$
Expected Number of Associations	0.1	0.9	5.9	96	360
% of Variance Explained	0.0	0.1	0.3	4.1	11.1

Case-Control Ratio 1:2

**Equal, 870 Causal Loci**	$n = 6387$	$n = 12500$	$n = 20000$	$n = 50000$	$n = 100000$
Expected Number of Associations	0.2	2.3	16.2	391	854
% of Variance Explained	0.0	0.1	0.5	11.8	25.8
**Uniform, 1230 Causal Loci**	$n = 6387$	$n = 12500$	$n = 20000$	$n = 50000$	$n = 100000$
Expected Number of Associations	0.2	2.7	18.3	331	759
% of Variance Explained	0.0	0.1	0.7	11.0	21.9
**Exponential, 2160 Causal Loci**	$n = 6387$	$n = 12500$	$n = 20000$	$n = 50000$	$n = 100000$
Expected Number of Associations	0.2	3.5	19.2	218	627
% of Variance Explained	0.0	0.2	1.0	7.8	16.3
**Chi Squared, 3390 Causal Loci**	$n = 6387$	$n = 12500$	$n = 20000$	$n = 50000$	$n = 100000$
Expected Number of Associations	0.3	3.6	19.2	198	567
% of Variance Explained	0.0	0.2	1.0	7.3	14.8

Table S6. Expected success of marginal (single-SNP) analyses. Our dataset had sample size $n = 6387$. Considering four distributions of heritability across causal loci, in each case supposing our point estimate for the number of causal variants is correct, we predict how the success of future genome-wide association studies will improve by increasing n. We also consider if the case-control ratio (currently 1258:5129) was instead increased to 1:2.

Pairing 1	Pairing 2	ρ	$P_{\rho=1}$	$P_{\rho=0}$
1958 BC vs Focal	NBS vs Non-Focal	0.56 (0.22)	0.007	0.001
NBS vs Focal	1958 BC vs Non-Focal	0.34 (0.18)	0.00002	0.02

Table S7. Bivariate analysis for focal and non-focal epilepsy. GCTA can estimate the correlation ρ between effect sizes for pairs of traits (here, two subtypes of epilepsy). To perform the test, it is necessary to pair each of the two sets of cases (here, focal or non-focal patients) with a control dataset (either National Blood Service or 1958 Birth Cohort), so we consider both possible combinations. We report the estimate of ρ (standard deviation in parentheses), P from a test that the two traits have identical genetic architectures ($\rho = 1$) and P from a test that the genetic architectures have no shared component ($\rho = 0$). In the main text, we report the average correlations and p-values across the two combinations.
